# Gankyrin drives malignant transformation of chronic liver damage-mediated fibrosis via the Rac1/JNK pathway

**DOI:** 10.1038/cddis.2015.120

**Published:** 2015-05-07

**Authors:** X Zhao, J Fu, A Xu, L Yu, J Zhu, R Dai, B Su, T Luo, N Li, W Qin, B Wang, J Jiang, S Li, Y Chen, H Wang

**Affiliations:** 1 Model Animal Research Center and MOE Key Laboratory of Model Animal for Disease Study, Nanjing University, Nanjing 210061, China; 2 International Cooperation Laboratory on Signal Transduction, Eastern Hepatobiliary Surgery Institute/Hospital, Shanghai 200438, China; 3 National Center for Liver Cancer, Shanghai 200438, China; 4 MOE Key Laboratory of Bioinformatics and Bioinformatics Division, TNLIST, Department of Antomation, Tsinghua University, Beijing 100084, China; 5 State Key Laboratory of Oncogenes and Related Genes, Cancer Institute of Renji Hospital, Shanghai Jiaotong University, Shanghai 200032, China

## Abstract

Hepatocarcinogenesis is a complex process involving chronic liver injury, inflammation, unregulated wound healing, subsequent fibrosis and carcinogenesis. To decipher the molecular mechanism underlying transition from chronic liver injury to dysplasia, we investigated the oncogenic role of gankyrin (PSMD10 or p28^GANK^) during malignant transformation in a transgenic mouse model. Here, we find that gankyrin increased in patients with cirrhosis. In addition to more severe liver fibrosis and tumorigenesis after DEN plus CCl_4_ treatment, hepatocyte-specific gankyrin-overexpressing mice (gankyrin^hep^) exhibited malignant transformation from liver fibrosis to tumors even under single CCl_4_ administration, whereas wild-type mice merely experienced fibrosis. Consistently, enhanced hepatic injury, severe inflammation and strengthened compensatory proliferation occurred in gankyrin^hep^ mice during CCl_4_ performance. This correlated with augmented expressions of cell cycle-related genes and abnormal activation of Rac1/c-jun N-terminal kinase (JNK). Pharmacological inhibition of the Rac1/JNK pathway attenuated hepatic fibrosis and prevented CCl_4_-induced carcinogenesis in gankyrin^hep^ mice. Together, these findings suggest that gankyrin promotes liver fibrosis/cirrhosis progression into hepatocarcinoma relying on a persistent liver injury and inflammatory microenvironment. Blockade of Rac1/JNK activation impeded gankyrin-mediated hepatocytic malignant transformation, indicating the combined inhibition of gankyrin and Rac1/JNK as a potential prevention mechanism for cirrhosis transition.

Hepatocellular carcinoma (HCC) is the prototype of inflammation-associated cancer, as most patients with HCC have an established background of unresolved chronic liver disease and cirrhosis.^1^ Major HCC risk factors include infection with hepatitis viruses, intake of aflatoxin-contaminated food, alcoholic liver disease, nonalcoholic steatohepatitis (NASH), chronic hepatic inflammation and cirrhosis.^2, 3^ Cirrhosis is the primary risk factor for developing HCC, accompanied by long periods of chronic liver disease.^4^ However, the molecular mechanisms of this malignant transformation remain elusive.

Gankyrin was identified as an oncoprotein that frequently overexpressed in human liver cancers and increased in the earlier stage of liver carcinogenesis.^5, 6^ It controls phosphorylated Rb and p53 degradation,^7, 8^ promotes the expansion of tumor-initiating cells^9^ and accelerates HCC progression. In addition, it also has been shown to regulate NF-*κ*B and AKT.^10, 11^ We recently found that, in a rat model of carcinogen-induced liver carcinogenesis, gankyrin elevated in the stage of cirrhosis.^12^ However, it is still unknown how gankyrin promotes hepatocarcinogenesis *in vivo*.

Here, we show increased gankyrin expression in patients with cirrhosis. We further used hepatocyte-specific gankyrin-overexpressing mice to study the role of gankyrin in hepatocarcinogenesis. Sustained gankyrin activation promotes DEN plus carbon tetrachloride (CCl_4_)-induced HCC formation. Moreover, it aggravates CCl_4_-mediated liver injury, hepatic fibrosis and ultimately leads to the development of cirrhosis and progression into HCC.

## Results

### Gankyrin accelerates hepatocarcinogenesis in livers with chronic damage and fibrosis

**Figure 1:**
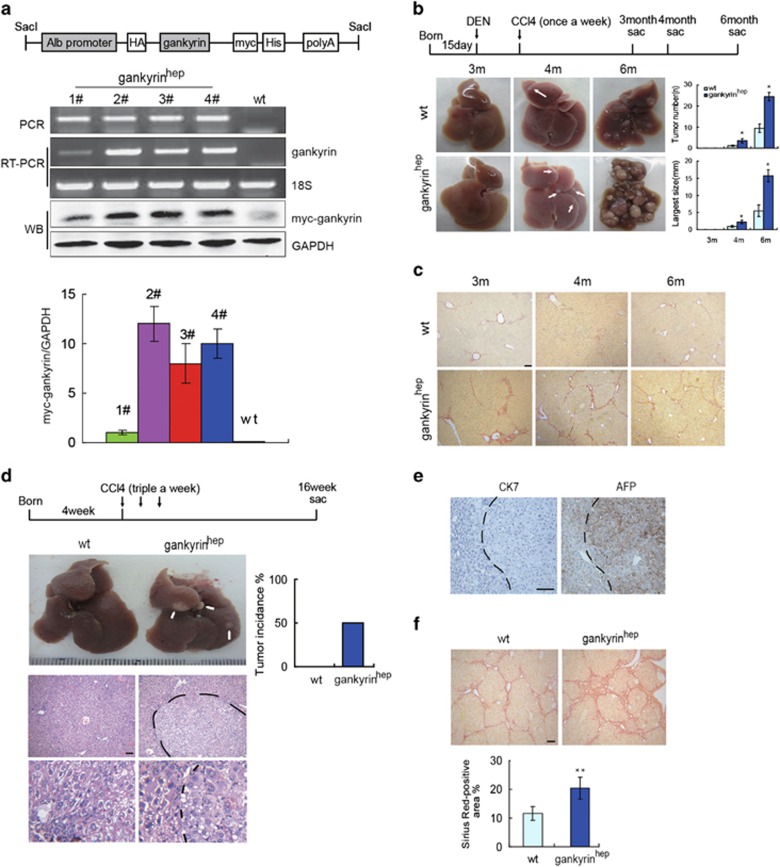
Gankyrin accelerates hepatocarcinogenesis in chronically injured liver. (**a**) Construct design of generating hepatocyte-specific gankyrin transgenic mice. Genotype of mice was determined by PCR, and expression levels of gankyrin in four different transgenic lines were examined by RT-PCR and western blot assay, normalized to GAPDH. (**b**) Macroscopic liver appearance of gankyrin^hep^ mice and littermates injected with DEN+CCl_4_ for different durations. Tumor number and the largest size were quantified (*n*=6). (**c**) Liver sections from **b** were collected for Sirius red staining ( × 100). (**d**) Representative photographs and H&E staining (original magnification × 100 and × 400) of livers from 16-week CCl_4_-treated gankyrin^hep^ mice (*n*=10) and control littermates (*n*=8). Tumor incidence was determined. (**e**) Immunohistochemical staining of CK7 and AFP in tumor area. (**f**) Sirius red staining of liver sections from **d** ( × 100) and quantified with the ImageJ software. Data are represented as mean±S.D. *P<0.05; **P<0.01. Scale bar, 100 *μ*m

Most patients with HCC have liver cirrhosis, which develops for long periods of chronic liver disease. Here, we observed elevated expression of gankyrin in clinical cirrhosis biopsies (Supplementary Figure S1), consistent with our previous studies that gankyrin gradually increased during DEN-induced liver cirrhosis and HCC in rat.^13^ Increased expression of gankyrin in human HCC correlated with poor survival and disease recurrence after surgery.^11^ To determine the contribution of gankyrin to hepatocarcinogenesis *in vivo*, we constructed a new transgenic mouse strain gankyrin^hep^, in which a human gankyrin protein expresses specifically in the hepatocytes driven by the albumin promoter. To examine the efficiency of gankyrin overexpression in the liver, we measured gankyrin expression in four different transgenic lines with RT-PCR and western blot assay. As shown in Figure 1a, the expression efficiency of gankyrin was variable in different lines, and thus we chose lines 2# and 4# for breeding and for further use. We subjected transgenic mice and littermates to a combination of DEN and the hepatotoxin CCl_4_ for 6 months. This model shares typical features of the majority of human HCC development, and it faithfully recapitulates the natural history of chronic injury, inflammation and fibrosis. Gankyrin^hep^ mice displayed a profound augment of tumor number, the largest size and hepatic fibrosis after DEN plus CCl_4_ injections (Figures 1b and c). Moreover, in the wild-type mice treated with DEN and CCl_4_, the endogenous gankyrin expression increased significantly (Figure 1d). Thus, we proposed that gankyrin accelerates chronic liver injury-induced hepatocarcinogenesis.

To further study whether gankyrin-directed tumorigenesis is associated with chronic hepatic injury, inflammation and fibrosis, a model of long-term CCl_4_ administration was used. We treated gankyrin^hep^ mice and control littermates with persistent CCl_4_ injection for 16 weeks. Expression of CYP2E1 was similar in liver tissues from control and gankyrin^hep^ mice, suggesting no difference between the two groups on CCl_4_ metabolism (Supplementary Figure S3). As illustrated in Figure 1e, nearly 50% of gankyrin^hep^ mice had developed single or multiple surface nodular tumors after 16 weeks of CCl_4_ treatment, whereas no nodules were observed in the surface of livers in the wild-type littermates. Histologic analysis revealed the occurrence of hepatocarcinoma only in gankyrin^hep^ mice (Figure 1e). Immunostaining with anti-alpha fetoprotein (AFP) antibody confirmed that the tumors of gankyrin^hep^ mice definitely originated from the hepatocytes. By contrast, no staining of CK7 was detected in the tumors (Figure 1f). Consistently, Sirius red staining showed more severe liver fibrosis in gankyrin^hep^ mice (Figure 1g). Thus, it suggests that gankyrin-induced hepatocarcinogenesis is associated with chronic liver injury and fibrosis.

### Gankyrin in hepatocytes enhances CCl_4_-mediated liver fibrosis

**Figure 2:**
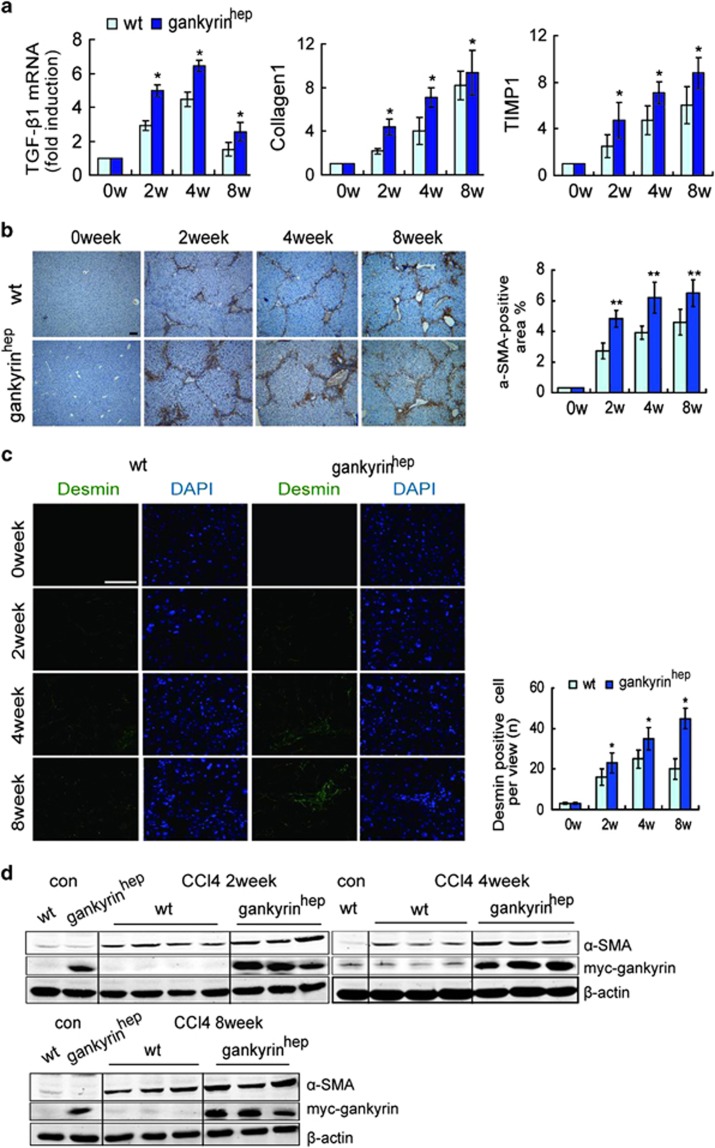
Overexpression of gankyrin causes more evident liver fibrosis and HSC activation upon chronic CCl_4_ challenges. (**a**) Mice were treated with CCl_4_ for 2, 4 and 8 weeks (*n*=6). TGF-*β*1, collagen-1 and TIMP1 mRNA expression were analyzed by real-time PCR, normalized to *β*-actin. (**b**) Immunostaining of *α*-SMA in the liver sections from **a** and quantifications. (**c**) Representative immunofluorescence staining of liver sections from **a** with combinations of desmin and DAPI, quantified by counting positive cells in 10 high-power fields. (**d**) Western blot assay for expression of *α*-SMA. Data are represented as mean±S.D. *P<0.05; **P<0.01. Scale bar, 100 *μ*m

Owing to a causal relationship between chronic injury, inflammation and hepatocarcinogenesis in most patients with HCC, the role of gankyrin during this process remains unknown. As presented in Supplementary Figure S4, after 2-, 4- and 8-week CCl_4_ treatments, gankyrin^hep^ mice had accelerated hepatic fibrosis, as evidenced by higher Sirius red staining. Consistently, real-time PCR analysis also confirmed higher expression of liver fibrosis-related genes TGF-*β*1, collagen-1 and TIMP1 in the liver from gankyrin^hep^ mice than that from littermates (Figure 2a). Given that hepatic stellate cells (HSCs) have a critical role in inducing fibrosis, we measured the levels of *α*-smooth muscle actin (*α*-SMA) and desmin proteins. As shown in Figures 2b–d, more *α*-SMA and desmin were displayed in the gankyrin group after CCl_4_ treatment. These results thereby demonstrated the importance of gankyrin in accelerating CCl_4_-mediated chronic liver fibrosis.

### Gankyrin augments CCl_4_-mediated chronic hepatic injury, inflammation and compensatory proliferation

**Figure 3:**
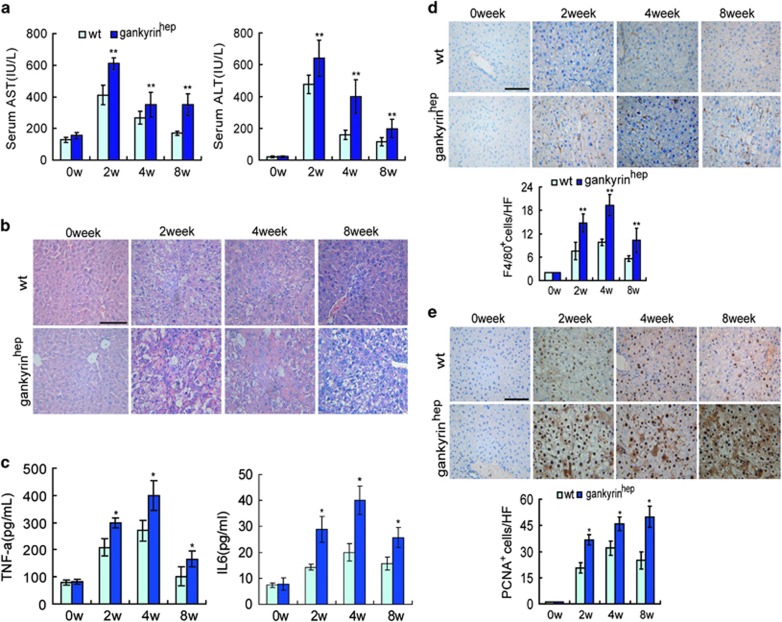
Gankyrin^hep^ mice are more susceptible to CCl_4_-induced liver injury, inflammatory response and compensatory proliferation. (**a**) Serum AST and ALT levels from livers (*n*=6) in Figure 2 were determined at 48 h after the last CCl_4_ injection. (**b**) H&E staining ( × 400) of liver sections from **a**. (**c**) ELISA analysis of serum levels of TNF-*α* and IL-6. (**d**) Immunostaining of F4/80, quantified by counting positive cells in 10 high-power fields (*n*=6). (**e**) Immunostaining of PCNA, quantified by counting positive cells in 10 high-power fields. *n*=6; data are represented as mean±S.D. *P<0.05; **P<0.01. Scale bar, 100 *μ*m

To reveal the basis for the increased susceptibility of gankyrin^hep^ mice to chemical carcinogenesis, we explored early effects of CCl_4_ on cell behavior and signal transduction. Higher levels of serum alanine transaminase (ALT) and aspartate transaminase (AST) indicate accelerated hepatocellular damage in gankyrin^hep^ mice (Figure 3a). As expected, morphologic analysis showed more abnormal architecture in gankyrin^hep^ mice relative to littermates after CCl_4_ treatment (Figure 3b). Moreover, more severe inflammatory response was observed in gankyrin^hep^ mice, as evidenced by higher serum levels of TNF-*α* or IL-6 and enhanced immunostaining of F4/80 (Figures 3c and d).

Owing to the high regenerative capacity of the liver, even when some fractions of hepatocytes undergo cell death in response to a carcinogenic dose of DEN or CCl_4_, the remaining surviving hepatocytes should undergo a compensatory proliferative response. Immunostaining of proliferating cell nuclear antigen (PCNA) displayed higher numbers of proliferating hepatocytes in gankyrin^hep^ mice than in the controls after CCl_4_ exposure (Figure 3e). Accordingly, microarray assay revealed significant upregulation or downregulation of a large majority of cell cycle genes in gankyrin^hep^ mice (data not shown), indicating that gankyrin further promoted hepatic compensatory proliferation after CCl_4_ treatment.

### Gankyrin enhances CCl_4_-mediated acute hepatocellular death via increasing JNK activity

We further determined an involvement of liver cell death in the process of gankyrin-directed hepatic injury. As shown in Supplementary Figures S5A and B, gankyrin^hep^ mice had grave heptocelluar damage owing to elevation of serum ALT and AST within 48 h after CCl_4_ treatment for 2 weeks, and more extensive aberrant architecture and necrotic cells. Immunoblotting showed more cleaved PARP, caspase-3 and cytochrome *c* in gankyrin^hep^ mice after CCl_4_ treatment (Supplementary Figure S5C). Accordingly, TUNEL assays revealed severe hepatocyte apoptosis in CCl_4_-treated gankyrin^hep^ mice relative to similarly treated control mice (Supplementary Figure S5D). Taken together, it indicated that hepatic gankyrin promotes CCl_4_-mediated hepatocyte death, which possibly resulted in subsequent fibrosis and tumor formation.

**Figure 4:**
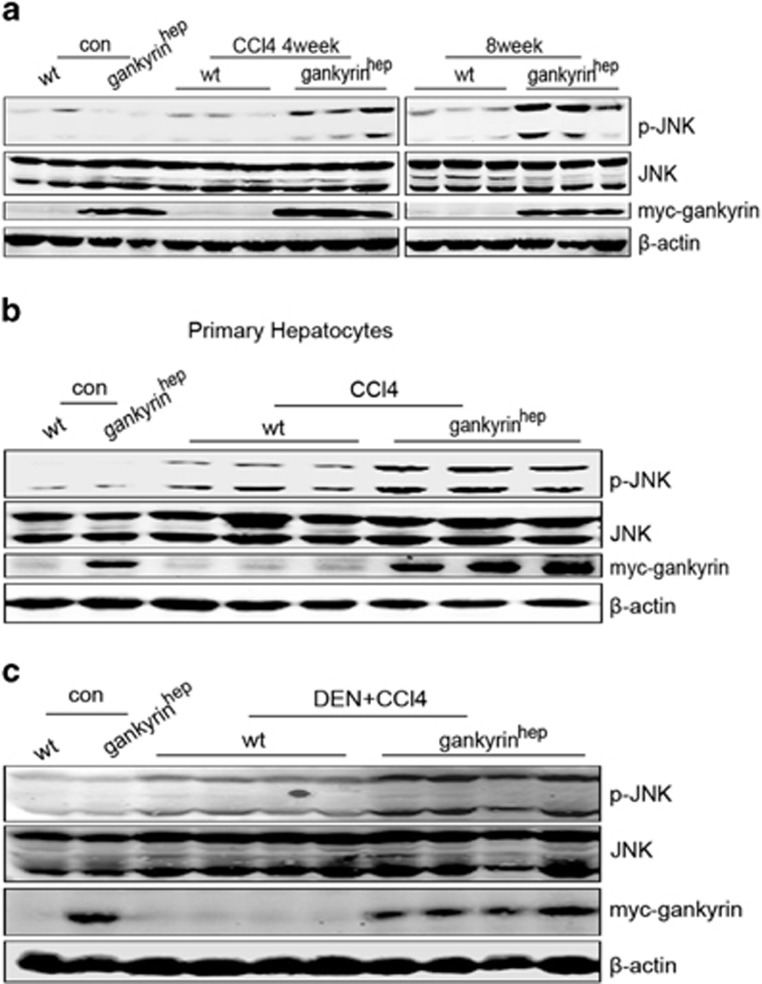
Gankyrin promotes acute CCl_4_-induced liver cell death via sustained JNK activation. (**a**) Western blot analysis of p-JNK and total JNK in 4-week and 8-week CCl_4_-treated mouse liver. (**b**) Primary hepatocytes isolated from olive oil or 4-week CCl_4_-treated control littermates or gankyrin^hep^ mice were subjected to western blot analysis. (**c**) Western blot analysis of p-JNK in the livers of DEN+CCl_4_-treated mice

To explore the molecular mechanism underlying the enhanced liver injury and tumorigenesis in gankyrin^hep^ mice, we detected several typical pathways involved in carcinogenesis. Although CCl_4_ induced AKT, ERK and p38/MAPK activation, no differences were found between gankyrin^hep^ mice and control littermates (Supplementary Figure S6). Phospho-JNK, a major contributor to acute liver failure and hepatocarcinogenesis,^14, 15, 16^ was observed to be constantly increased in the gankyrin group after CCl_4_ treatment. By contrast, it transiently elevated to the top within 12 h but lowered then in the control group (Figure 4a; Supplementary Figure S7). Moreover, enhanced phospho-JNK activity was detected in the primary hepatocytes isolated from 4-week CCl_4_-treated gankyrin^hep^ mice (Figure 4b) or in the liver tumors from DEN plus CCl_4_-treated gankyrin^hep^ mice (Figure 4c). Thus, it suggests that persistent JNK activation most likely contributes to gankyrin-induced liver injury and tumorigenesis after CCl_4_ exposure.

### Gankyrin enhanced JNK activation via the Rac1 pathway

**Figure 5:**
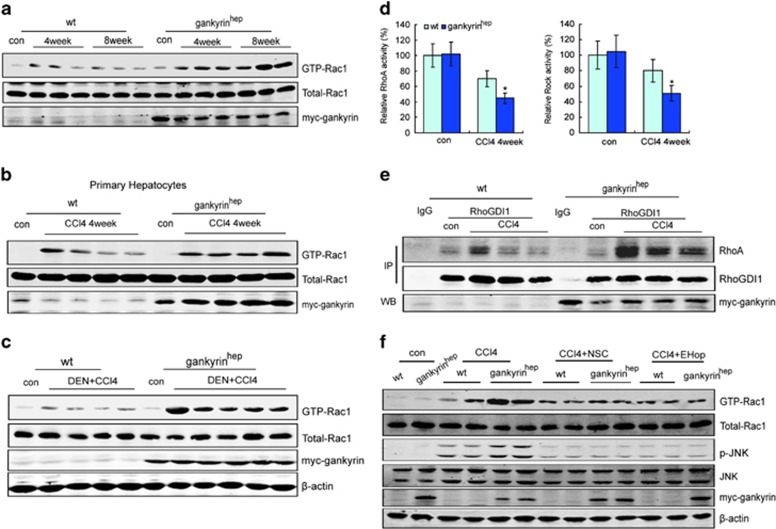
Gankyrin increased JNK activation via Rac1. (**a**) Rac1 activity was assayed in liver tissues after a 4-week and an 8-week CCl_4_ treatment. (**b**) Primary hepatocytes in mice with olive oil or 4-week CCl_4_ treatment were used for Rac1 activity assay. (**c**) Liver samples from DEN+CCl_4_-treated mice were collected for Rac1 activity assay. (**d**) RhoA or ROCK activity assay was analyzed in olive oil or 4-week CCl_4_-treated mouse livers. (**e**) Immunoprecipitation with RhoGDI1 antibody in mouse livers from **d**. (**f**) Liver samples from both groups treated with olive oil, CCl_4_, CCl_4_+NSC23766 or +EHop-016 for 4 weeks were collected for Rac1 activity assay and western blot analysis. Data are represented as mean±S.D. *P<0.05

Small GTPases including RhoA, Rac1 and Cdc42 are able to trigger MAPK signaling leading to apoptosis in various cells.^17, 18^ Rac1 activation has been reported to induce apoptosis through activating the JNK pathway.^19, 20^ In the present study, CCl_4_ treatment for different time points resulted in further elevation and persistence of Rac1 activity in gankyrin^hep^ mice, whereas a relatively weak and transient rise was observed in the littermate group (Figure 5a; Supplementary Figure S8A). Moreover, increased Rac1 activity was also detected in the primary hepatocytes of the 4-week CCl_4_-treated gankyrin^hep^ group (Figure 5b). Consistently, DEN+CCl_4_-treated gankyrin^hep^ group also displayed higher Rac1 activity than littermate control did (Figure 5c).

RhoGDI directly interacts with Rac1 as a cytosolic inhibitor.^21^ Here, through coimmunoprecipitation in liver tissues from gankyrin^hep^ or littermate control, the association of RhoGDI1 with Rac1 decreased gradually within 48-h CCl_4_ administration, but their interaction was not altered by gankyrin (Supplementary Figure S8B). Thus, it indicated no effect of gankyrin on interaction between RhoGDI1 and Rac1.

Gankyrin has been reported to activate Rac1 through the RhoA/ROCK pathway.^22, 23^ To explore the possibility of RhoA/ROCK involved in gankyrin-mediated Rac1 activation, we then examined the RhoA and ROCK activity in both groups. As displayed in Figure 5d, RhoA and ROCK activities markedly decreased after CCl_4_ treatment, and further lowered in the gankyrin^hep^ group. Because of RhoGDI as a pivotal inhibitor of RhoA,^24^ we next investigated the effect of gankyrin on RhoGDI1 inhibition on RhoA. Immunoprecipitation showed a more enhanced connection between RhoGDI1 and RhoA in gankyrin^hep^ mice than that in littermate ones (Figure 5e). Collectively, it suggested that gankyrin promoted the interaction between RhoGDI and RhoA and inhibited RhoA/ROCK activity, leading to increased Rac1 activity.

**Figure 6:**
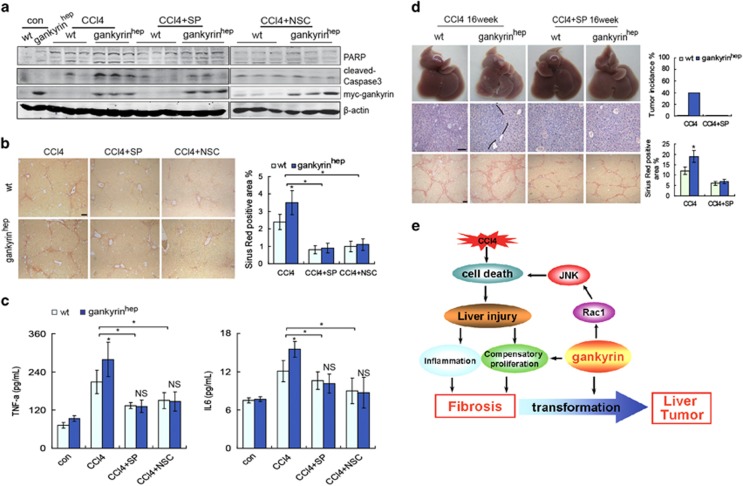
JNK or Rac1 inhibitor improved gankyrin-mediated liver fibrosis and prevented gankyrin-induced carcinogenesis after CCl_4_ management. (**a**)Gankyrin^hep^ and littermate mice were injected with CCl_4_, CCl_4_+SP600125 or CCl_4_+NSC23766 for 4 weeks (*n*=6). Liver samples were collected for immunoblot for PARP and cleaved caspase-3. (**b**) Liver sections in **a** were subjected to Sirius red staining. (**c**) Serum from **a** was analyzed by ELISA for TNF-*α* or IL-6. (**d**) Representative images of liver sections, H&E and Sirius red staining of 16-week CCl_4_ or CCl_4_+SP600125-treated mice. Tumor incidence and Sirius red-positive staining were quantified. Data are represented as mean±S.D. *P<0.05. Scale bar, 100 *μ*m. (**e**) Schematic representation of the potential mechanism of gankyrin in liver malignant transformation

To further confirm the involvement of Rac1 activity in gankyrin-mediated JNK activation, two small-molecule inhibitors for Rac1, NSC23766 or EHop-016, were used. As shown in Figure 6f, treatment with NSC23766 or EHop-016 reduced Rac1 activity *in vivo* after CCl_4_ administration. Phospho-JNK levels in the gankyrin^hep^ group declined much more closely to that in controls (Figure 5f), directing that gankyrin sustained and enhanced JNK activity via the Rac1 pathway in this model.

### JNK or Rac1 inhibitors ameliorated gankyrin-mediated liver fibrosis and blocked carcinogenesis after CCl_4_ exposure

As gankyrin^hep^ mice displayed persistent Rac1/JNK activation during CCl_4_ handling, it seems likely to account for the elevated susceptibility of gankyrin^hep^ mice to CCl_4_-induced liver fibrosis and hepatocarcinogenesis. To test this possibility, we treated both groups with 4-week CCl_4_ in the presence or absence of specific JNK or Rac1 inhibitors, SP600125 or NSC23766, respectively. First, CCl_4_-mediated hepatocyte apoptosis in gankyrin^hep^ mice was improved by SP600125 or NSC23766, evidenced by decreased cleavage of PARP and caspase-3 (Figure 6a). Moreover, both the liver fibrosis masses and inflammation degrees in gankyrin^hep^ mice after CCl_4_ treatment were obviously ameliorated to that in control mice (Figures 6b and c). Correspondingly, after SP600125 treatment for 3 months, 16 weeks of CCl_4_ treatment-induced liver cancers in gankyrin^hep^ mice was completely blocked, and liver cirrhosis was improved as well (Figure 6d). Meanwhile, elevated serum AST and ALT in gankyrin^hep^ mice was reversed by the SP600125 treatment. In addition, the augmented inflammation and hepatocellular proliferation in the gankyrin^hep^ group after 16-week CCl_4_ treatment were also restricted significantly by SP600125 (Supplementary Figures S10A and B). Taken together, it implied that Rac1/JNK activation accounts for gankyrin-mediated liver carcinogenesis in mice after CCl_4_ treatment.

## Dicussion

HCC carcinogenesis is a complex process involving various etiologic factors, dysregulations of several signal pathways, as well as genetic alterations, and it ultimately results in malignant transformation and HCC progression.^25, 26^ To date, many investigations have focused on the possible mechanisms involved in liver tumorigenesis. However, mediators that are responsible for the high risk to develop HCC in the chronically injured liver are largely unknown.

One of the key features of gankyrin as an oncogene is its dysregulation of the balance of proliferation and apoptosis. However, its role in carcinogenesis *in vivo* remains unclear. Given that gankyrin increased in patients with cirrhosis, or at earlier stages of DEN-induced HCC formation in a rat model, it very likely induces hepatocarcinogenesis. In our study, we did not find any difference of tumor formation between the two groups under a single DEN treatment (Supplementary Figure S2), consistent with Fujita recent reports.^27^ However, DEN plus CCl_4_ treatment resulted in more dysplastic nodules in gankyrin^hep^ mice than in littermates. This apparent contradiction can be explained by the features of the murine model of DEN-induced HCC; only one single postnatal injection is performed, which cannot reflect the multistage hepatocarcinogenesis because the successsion leading to continuing DNA damage, fibrosis, cirrhosis and tumor is entirely skipped. In contrast, the model of a combination of DEN and CCl_4_ incorporates chronic injury, inflammation, fibrogenesis and thus faithfully recapitulates human HCC development. Thus, the oncogenic function of gankyrin mostly relied on persistent hepatocyte damage and inflammatory microenvironment.

Chronic inflammation is a critical contributor to carcinogenesis, including HCC, colonal cancer and lung cancer.^28, 29, 30^ CCl_4_ administration produces the reactive metabolite trichloromethyl radical via cytochrome *P*450, and it causes hepatocellular injury and necrosis by eliciting the production of reactive oxygen intermediates and by peroxidative degradation of membrane phospholipids.^31^ Repeated administration of CCl_4_ for a prolonged period of over 2 years induces 50% HCC in mice.^32, 33^ Intriguingly, after a single prolonged injection of CCl_4_ for 16 weeks, satellite nodules appeared only on the livers of gankyrin^hep^ mice. Moreover, tumors developing by this regimen showed typical features of HCC. In contrast, under the same treatment, the littermate control only underwent the development of cirrhosis but not malignant transformation. In addition to cell death, more proliferation of hepatocytes in gankyrin^hep^ mice after CCl_4_ stimulation further confirmed the synergistic effect of gankyrin on compensatory proliferation of hepatocytes. Given its promotion of cell survival, it seems contradictory for gankyrin augmenting CCl_4_-induced hepatocyte deaths, which triggered more compensatory proliferative response in the parenchyma leading to enhanced tumor formation. However, the differences between *in vitro* and *in vivo* might be explained by the inflammatory component in experimental animal models. As compensatory proliferation is critical for tumor promotion, the most possible interpretation of our results is that, in hepatocytes, gankyrin promotes malignant transformation of liver cirrhosis by enhancing compensatory growth under inflammatory microenvironment.

Our previous studies demonstrated that gankyrin activates AKT signaling to promote HCC invasion and metastasis.^11^ However, no obvious phospho-AKT was observed in gankyrin^hep^ mice or in littermate control even after CCl_4_ exposure (Supplementary Figure S6A). Given the inhibitory effect of gankyrin on NF-*κ*B activity via nuclear export of RelA, we herein also observed fewer cells with nuclear localization of RelA in the gankyrin^hep^ group regardless of no impression on NF-*κ*B downstream genes relating to apoptosis, indicating that NF-*κ*B is probably dispensable for gankyrin-mediated hepatocarcinogenesis in the CCl_4_ model (Supplementary Figures S9A and B). Considering that gankyrin can regulate p53, Rb and CDK4 signals, related molecules were compared at the protein level, including p53 and its downstream targets Bax and p21, phospho-RB and CDK4 (Supplementary Figure S9C). However, almost no difference was found between the gankyrin^hep^ and control mice, indicating that these pathways are not involved. Moreover, no difference of Oct4 expression was found between the two groups after CCl_4_ administration for 8 weeks (Supplementary Figure S9D). It seems contradictory to our preceeding report that gankyrin expands liver tumor-initiator cells via Oct4.^9^ To our knowledge, one possible reason is that HSCs have a pivotal role to orchestrate parenchymal and nonparenchymal cells, rather than progenitor cells, involving this process. Of course, more details need to be determined in the future.

The JNK pathway has been implicated in regulating cellular stress response, apoptosis, malignant transformation and HCC carcinogenesis.^34, 35^ JNK activation for a long time will trigger apoptosis in some cell types.^36, 37, 38, 39^ Here, after DEN plus CCl_4_ or single CCl_4_ stimulation, persistent JNK activation displayed in liver tissues or in primary hepatocytes from gankyrin^hep^ mice, accompanied by increased apoptosis of liver cells. Small GTPase Rac1 is able to activate JNK to influence apoptotic cell death.^19^ In our present study, overexpression of gankyrin increased CCl_4_-induced Rac1 activity relying on RhoA, another member of the Rho GTPase family. It has been reported that RhoA/ROCK inhibits the Rac pathway and gankyrin activates Rac through the RhoA/ROCK pathway.^22, 23^ Consistently, RhoA/ROCK activity decreased, whereas the interaction between RhoDGI and RhoA increased in gankyrin^hep^ mice. Our postulation is that gankyrin enhances the association between RhoGDI and RhoA, inhibits RhoA activity and then indirectly activates the Rac/JNK signals. In addition, specific Rac inhibitors significantly blocked gankyrin-mediated JNK activation. Combined with impediment of gankyrin-mediated hepatic fibrosis and HCC formation by Rac or JNK inhibitor, Rac1 is very likely involved in gankyrin-induced JNK activation.

A procarcinogenic effect for JNK in the liver especially in nonparenchymal cells has been demonstrated, whereas its tumor suppressor role in hepatocytes has also been reported.^15^ The effect of gankyrin on JNK activity in nonparenchymal cells needs to be further explored in the future. Cirrhosis is the most common and unifying condition associated with hepatocarcinogenesis, with almost 90% of HCC cases arising in the setting of established cirrhosis, which develops after long latencies (20–40 years) of chronic liver disease.^3, 40^ At present, a range of primary treatment options including antiviral therapy and weight reduction strategies are variably effective in these conditions. Unfortunately, a significant number of patients still progress to end-stage liver disease and many require orthotopic liver transplantation. Thus, it is urgently required for developing new therapeutic strategies for the prevention or treatment of hepatic fibrosis, cirrhosis and HCC. Our results demonstrate the significance of Rac1/JNK in cirrhosis or HCC and the potential utilization of Rac1/JNK targeting for liver diseases therapy. Recently, a new strategy targeting multiple kinases was proposed for cancer treatment.^41, 42, 43^ Our previous findings have shown that targeting gankyrin significantly blocked tumor-initiating cell-mediated tumor cell expansion and metastasis.^9, 11^ In light of our studies, a combination of gankyrin and Rac1/JNK targeting may profoundly improve liver cirrhosis therapies.

## Materials and Methods

### Human tissue specimen

HCC tissue samples were obtained from surgical resections of liver tumors with informed consent of the patients at the Eastern Hepatobiliary Surgery Hospital (Shanghai, China). Cirrhosis tissues were obtained from patients after liver transplantation at Zhongshan Hospital, Fudan University (Shanghai, China). All human sample collection procedures were approved by the China Ethical Review Committee.

### Transgenic mice

Gankyrin^hep^ transgenic mice were generated as follows. A full-length cDNA of human gankyrin, followed by myc-tag and His-tag, was ligated into the pL253 vector after the 12.5-kb albumin promoter. The 13.6-kb whole-insert fragment was excised by SacI and injected into fertilized eggs. Mice were maintained on a C57BL/6 J background. Animals were housed on a standard rodent chow diet with 12-h light-dark cycle. Offspring were genotyped using the following primers gt-forward 5′-AGCGAGTCTTTCTGCACACA-3′, gt-reverse 5′-ACAGCATTCACTTGAGCACC-3′. All the experiments were carried out on two different transgenic lines.

### DEN or DEN+CCl_4_-induced liver cancer model

All animals received human care according to the criteria outlined in the ‘Guide for the Care and Use of Laboratory Animals' prepared by the National Academy of Sciences and published by the NIH (publication 86-23 revised 1985). The DEN-induced liver cancer model was established as described previously.^44^ In brief, 15-day-old gankyrin^hep^ mice and their littermates were injected with 25 mg/kg body weight of DEN (Sigma-Aldrich, St. Louis, MO, USA). After 9 months on normal chow, the mice were killed. In addition, the construction of the DEN+CCl_4_-induced liver tumor was described in the reports of Dianne H Dapito.^45^ For gankyrinhepmice and wild-type controls, HCC was induced by the combination of DEN (25 mg/kg i.p.) given at day 15 postpartum and weekly injections of CCl_4_ (5 ml/kg i.p., 10% dissolved in olive oil) for different time.

### CCl_4_-induced liver injury, fibrosis and tumor formation

Four- to six-week-old male gankyrin^hep^ mice and their wild-type littermates were injected with CCl_4_ (4 ml/kg i.p., 5% dissolved in olive oil) three times a week for up to 16 weeks.^46^ The mice were killed at different time points after the last injection of CCl_4_, and liver tissues were harvested for experiments.

### Inhibitor treatment

Specific JNK inhibitor, SP600125 (Selleck Chemicals, Houston, TX, USA), was injected at a dose of 1 mg/kg/day i.p. for 2 weeks or 1 mg/kg twice a week for 3 months. Rac1-specific small-molecular inhibitors, NSC23766 and EHop-016 (Selleck Chemicals), were injected intraperitoneally at a dose of 2 mg/kg/day for 4 weeks.

### Histology of mouse liver tissue

All paraffin-embedded liver tissues were stained with hematoxylin and eosin (H&E) for analysis of morphologic changes. Sirius red staining was used to determine collagen deposition. Immunohistochemistry and Sirius red staining were performed according to the routine protocol. The primary antibodies were as follows: gankyrin (Santa Cruz, Dallas, TX, USA), *α*-SMA (Sigma-Aldrich) and RelA (Santa Cruz). Apoptosis was assessed by TUNEL staining of paraffin-embedded slides (Calbiochem, La Jolla, CA, USA). Proliferation was assessed by immunostaining for PCNA (Cell Signaling Technology, Boston, MA, USA) staining.

### Measurement of transaminase activities

Serum ALT and AST levels were determined using a Fuji DRICHEM 55500 V (Fuji Medical System, Tokyo, Japan) according to the manufacturer's instructions.

### Western blot analysis

Human tissue specimen or whole mouse liver tissue was homogenized in Triton lysis buffer (20 mM Tris (pH 7.4), 137 mM NaCl, 10% glycerol, 1% Triton X-100 (Sigma-Aldrich), 2 mM EDTA, 1 mM PMSF, 10 mM NaF, 5 mg/ml aprotinin, 20 mM leupeptin and 1 mM sodium orthovanadate) and centrifuged at 12 000 r.p.m. for 15 min. Protein extracts were subjected to SDS-PAGE and analyzed using the following primary antibodies: gankyrin (Santa Cruz), *α*-SMA (Sigma-Aldrich), RhoGDI1 (Santa Cruz), RhoA (ABclonal, Wuhan, China), PARP, cleaved caspase-3, cytochrome *c*, p-JNK, JNK, p-AKT, AKT, p-ERK, ERK, p-P38, P38, Rac1 and *β*-actin (from Cell Signaling Technology). Secondary antibodies were labeled with IRDye 700 (Rockland Immunochemicals, Gilbertsville, PA, USA). Protein levels were detected by the Odyssey system (LiCor, Lincoln, NE, USA).

### Cytokine measurement in serum

Levels of TNF-*α* and IL-6 were measured with a commercial ELISA kit according to the instructions of the manufacturer (Dakewei, Shenzhen, China; Synergy 2 multi-mode microplate reader, BioTek, Winooski, VT, USA).

### Quantitative real-time PCR analysis

Real-time PCR was performed using the SYBR Green PCR kit (Applied Biosystems, Foster City, CA, USA) and ABI 7900HT fast real-time PCR system (Applied Biosystems). The following primers were used for gene expression: TGF-*β*1-forward 5′-GGTTCATGTCATGGATGGTGC-3′, TGF-*β*1-reverse 5′-TGACGTCACTGGAGTTGTACGG-3′ Colla1-forward 5′-GGAAACCTCTCTCGCCTCTT-3′, Colla1-reverse 5′-GAACAGGGTGTTCCTGAGA-3′; TIMP1-forward 5′-GGCTAAATTCATGGGTTCAC-3′, TIMP1-reverse 5′-CTCAGAGTACGCCAGGGAACCAAG-3′; Bcl-XL-forward 5′-GCTTAGCCCTTTTCGAGGAC-3′, Bcl-XL-reverse 5′-CCCACCAGGACTGGATAATG-3′ Bid-forward 5′-TTCTCCAAAGCTCTGGCTGT-3′, Bid-reverse 5′-GATGTCTGGCAATGTTGTGG-3′ XIAP-forward 5′-TTGGAACATGGACATCCTCA-3′, XIAP-reverse 5′-TGCCCCTTCTCATCCAATAG-3′ and Bcl2-forward 5′-GGTGGTGGAGGAACTCTTCA-3′, Bcl2- reverse 5′-ACCTACCCAGCCTCCGTTAT-3′.

### Rac1 activity assay

Rac1 activity assay was performed using the Active Rac1 Detection kit purchased from Cell Signaling Technology. Briefly, GST-PAK1-PBD fusion protein is used to bind the activated form of GTP-bound Rac1, which can then be immunoprecipitated with glutathione resin. Rac1 activation levels are then determined by western blot analysis using a Rac1 antibody.

### Primary hepatocyte isolation

Primary hepatocytes were isolated from untreated or CCl_4_-treated wild-type and gankyrin^hep^ mice using liver perfusion and isolation techniques, as previously described.^47^

### Statistical analysis

Data are expressed as mean±S.D. Student's *t*-test was performed to compare values from two groups. Statistical significance was taken at the *P*<0.05 level.

## Abbreviations

HCC: hepatocellular carcinoma
CCl_4_: carbon tetrachloride
DEN: diethylnitrosamine
CK7: cytokeratin-7
AFP: alpha fetoprotein
JNK: c-jun N-terminal kinase
*α*-SMA: *α*-smooth muscle actin
TUNEL: Terminal-deoxynucleotidyl transferase-mediated nick end labeling
PCNA: proliferating cell nuclear antigen
ALT: alanine transaminase
AST: aspartate transaminase
HSC: hepatic stellate cell
