# Supplementary material for: Gankyrin drives malignant transformation of chronic liver damage-mediated fibrosis via the Rac1/JNK pathway
Source: Cell Death Dis. 2015 May 7;6(5):e1751–. doi: 10.1038/cddis.2015.120 (PMC4669699; doi:10.1038/cddis.2015.120)
Supplement: Supplementary Information [file cddis2015120x1.doc]

**Gankyrin drives malignant transformation of chronic liver damage-mediated fibrosis via Rac1/JNK pathway**

Xiaofang Zhao, Jing Fu, An Xu, Lexing Yu, Junjie Zhu, Rongyang Dai, Bo Su, Tao Luo, Ning Li, Wenhao Qin, Bibo Wang, Jinhua Jiang, Shao Li, Yao Chen, Hongyang Wang

**Supplemental Figures**

**Figure S1.** Liver sections from human normal, cirrhosis and HCC tissues were detected by (A) immunohistochemistry (normal n=20, cirrhosis n=26, HCC n=19) and (B) immunoblot (normal n=26, cirrhosis n=25) for gankyrin, and quantified by Image J and Odyssey software. Protein expressions of gankyrin were normalized by β-actin.

**Figure S2.** Mice were injected with DEN at 15th day postnatal and sacrificed at 9 months after treatment. Representative pictures of livers from wild type and gankyrin^hep^ mice. The tumor number and size were examined. No statistically significant differences between wild type and gankyrin^hep^ mice were detected. NS, not significant. Student’s *t* test.

**Figure S3.** (A) Immunoblot of the endogenous gankyrin in the livers from wild type mice after DEN+CCl4 treatment. (B) Western blot of untreated or 16 week-CCl4 treated wild type and gankyrin^hep^ mice for expression of CYP2E1.

**Figure S4.** Mice were treated with CCl4 for 2, 4 and 8weeks (n=6). Sirius red staining and quantification.

**Figure S5.** (A) Gankyrin^hep^ mice and control littermates were injected with CCl4 for 2 weeks and sacrificed at indicated time (n = 6). Serum AST and ALT of mice were determined. (B) H&E staining of liver sections from (A), original magnification × 100 and × 400. (C) Total liver lysates from (A) were subjected to western blot analysis. (D) TUNEL staining of liver sections from 24 and 48 hours-sacrificed mice; quantified by counting positive cells in 10 high-power fields.

**Figure S6.** (A) Western blot of different time CCl4 treated wild type and gankyrin^hep^ mice liver samples for p-AKT, AKT. (B) Western blot for p-ERK, ERK, p-P38, P38.

**Figure S7.** Western blot analysis of p-JNK and total JNK in mice liver within 48hours after 2 week-CCl4 treatment.

**Figure S8.** (A) Rac1 activity was assayed in liver tissues of Figure S7. (B) Immunoprecipitation with RhoGDI1 antibody in above mice liver.

**Figure S9.** (A) Liver sections from 4 week-CCl4 treated wild type and gankyrin^hep^ mice were subjected to immunohistochemistry staining for gankyrin and RelA. (B) Bcl-xl, Bid, XIAP and Bcl2 mRNA expression in livers from olive oil or 4 week-CCl4 treated mice liver by real-time PCR. Normalized to β-actin. (C) OCT4 expression was evaluated by western blot. (D) Western blot of 4 week and 8week-CCl4 treated liver samples for p53, Bax, p21, p-RB, CDK4.

**Figure S10.** Serum ALT and AST (A), mRNA levels of TNF-α and IL-6, the immunostaining of F4/80 and PCNA (B) were evaluated in 16 week-CCl4 or CCl4+SP600125 treated mice.
