# Supplementary figures and images for: Gankyrin drives malignant transformation of chronic liver damage-mediated fibrosis via the Rac1/JNK pathway
Source: Cell Death Dis. 2015 May 7;6(5):e1751–. doi: 10.1038/cddis.2015.120 (PMC4669699; doi:10.1038/cddis.2015.120)

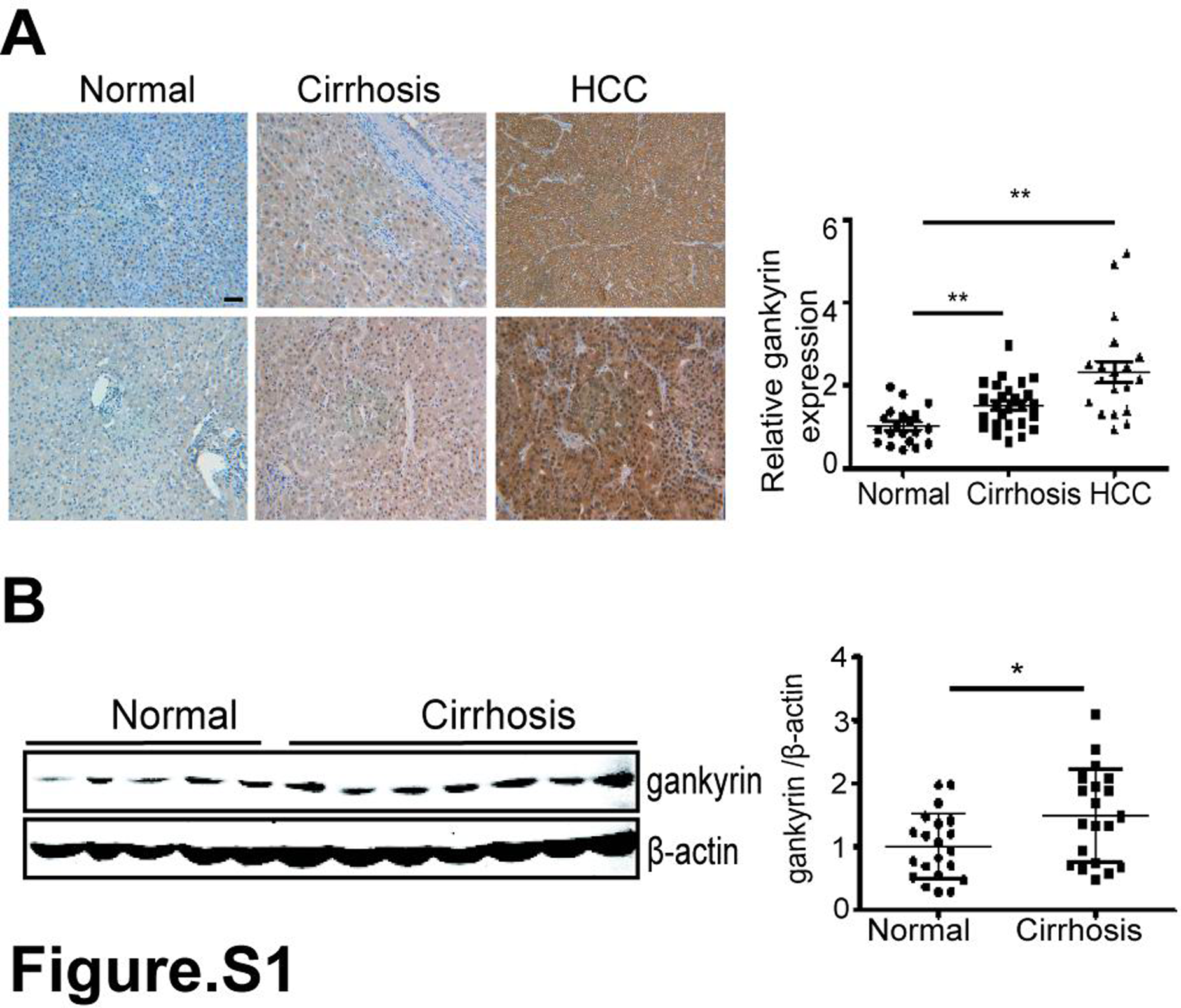

Supplement: Supplementary Figure 1 [file cddis2015120x2.tif]

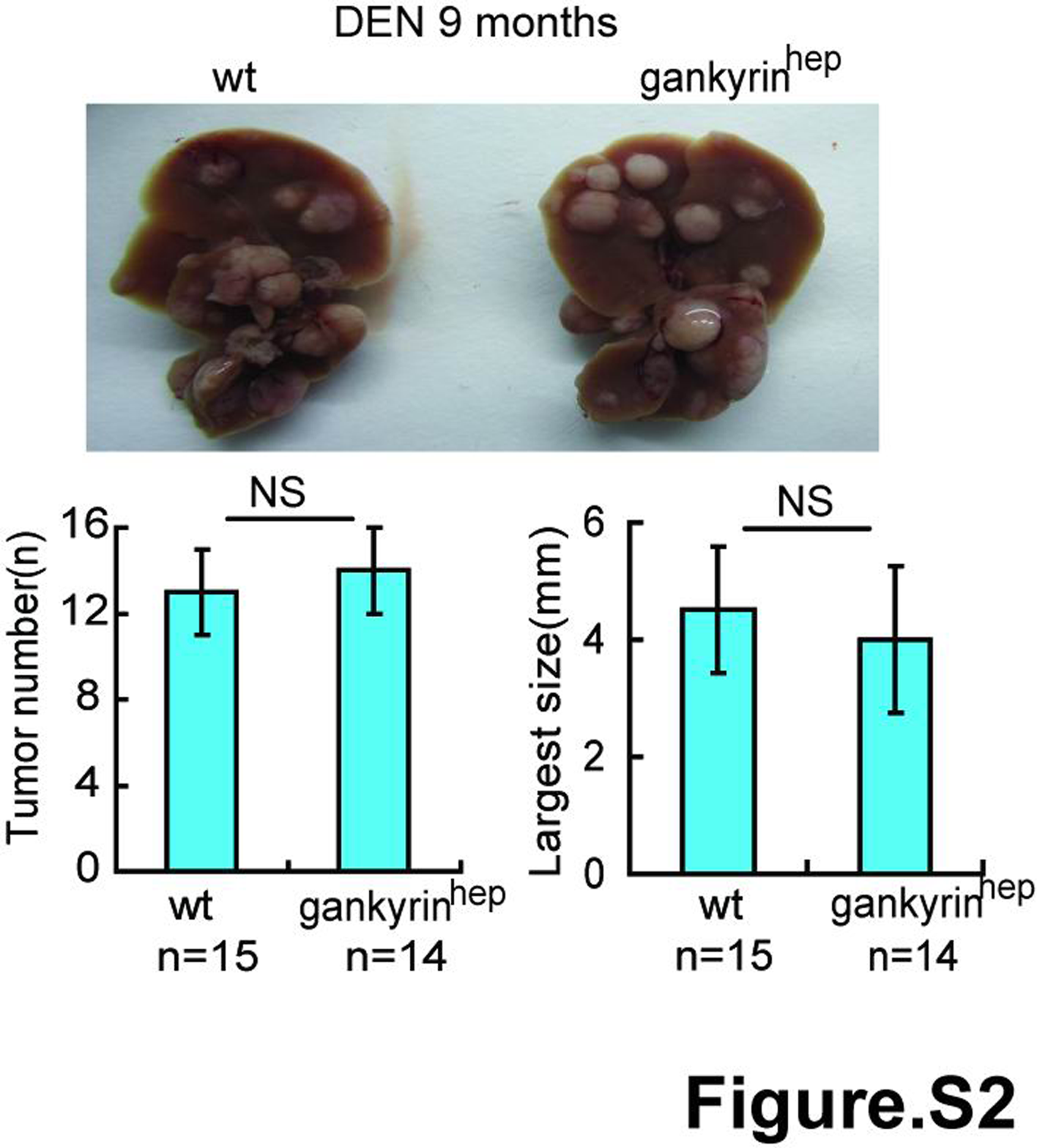

Supplement: Supplementary Figure 2 [file cddis2015120x3.tif]

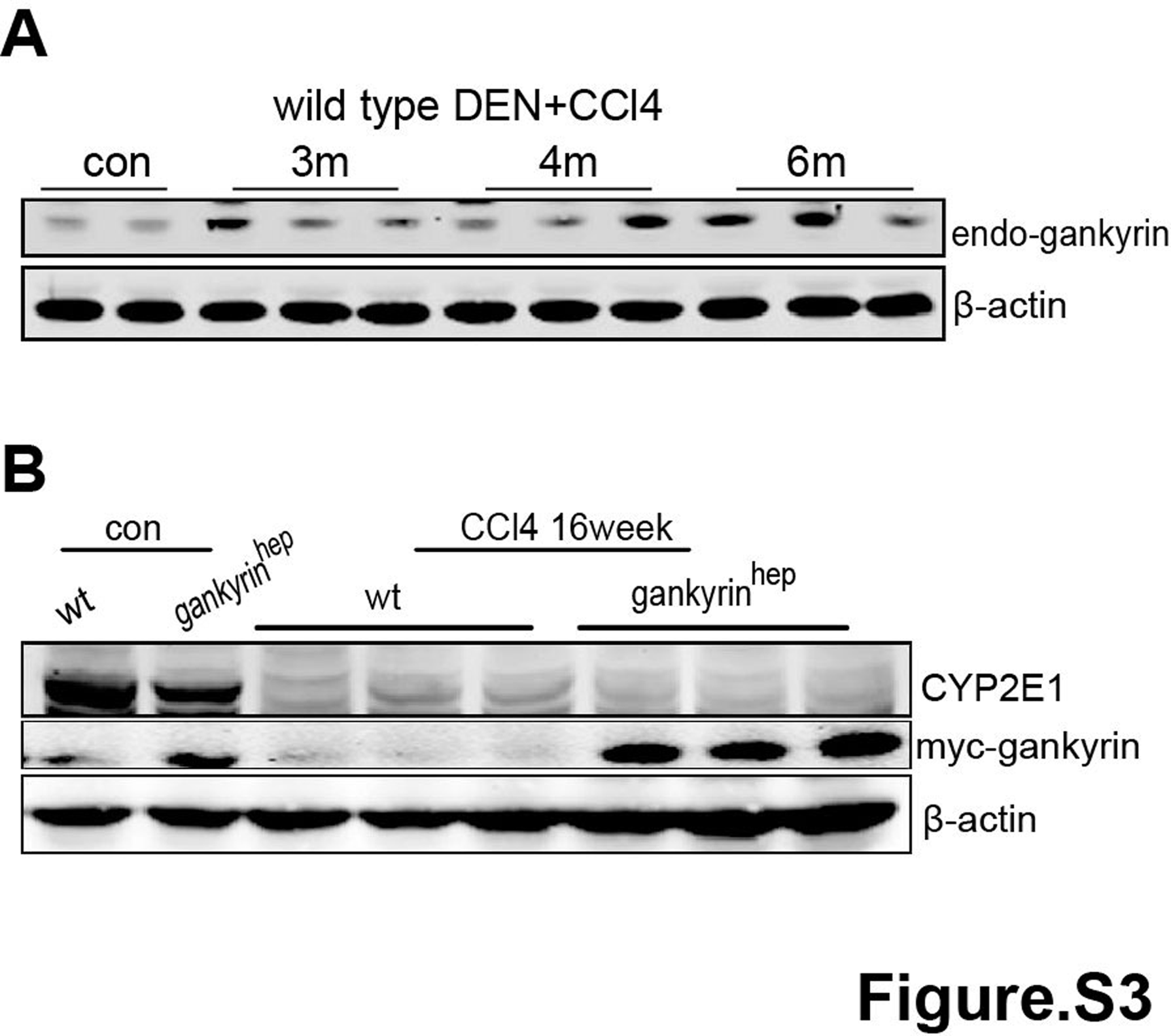

Supplement: Supplementary Figure 3 [file cddis2015120x4.tif]

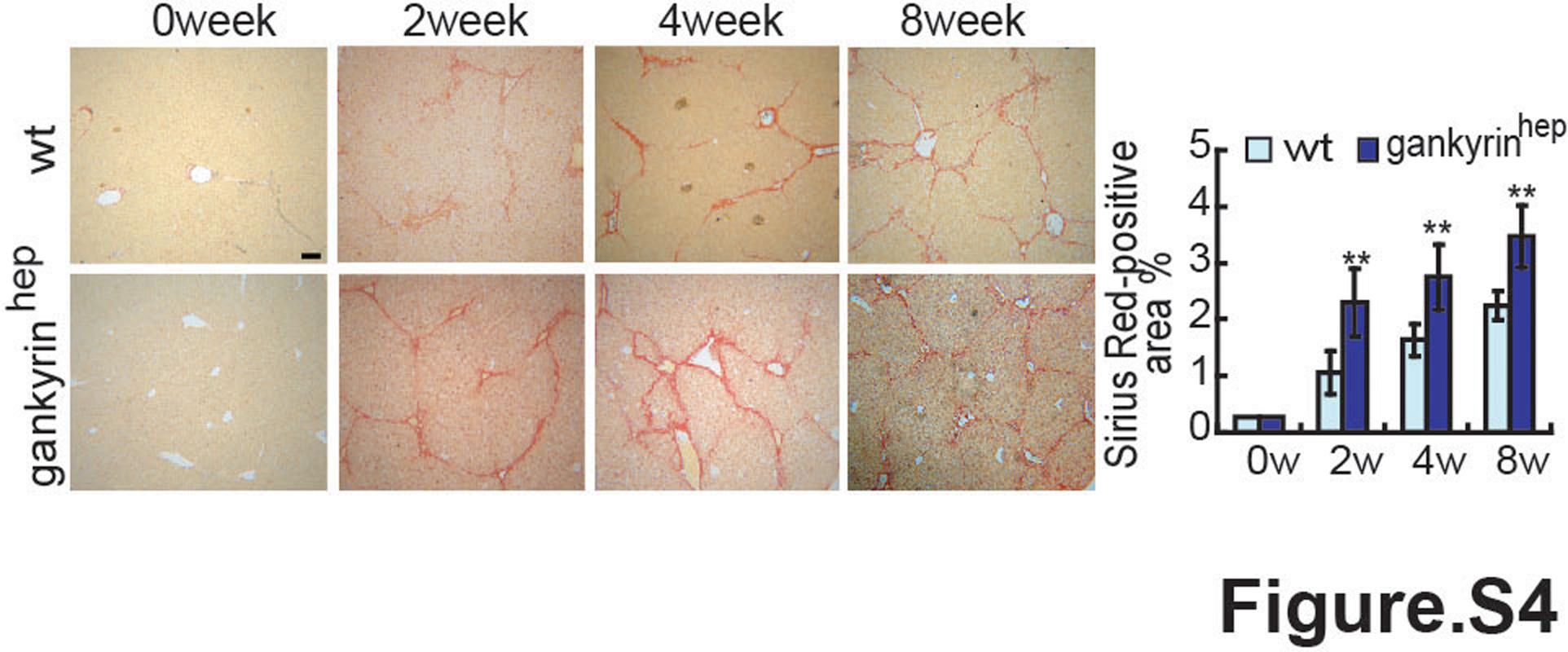

Supplement: Supplementary Figure 4 [file cddis2015120x5.tif]

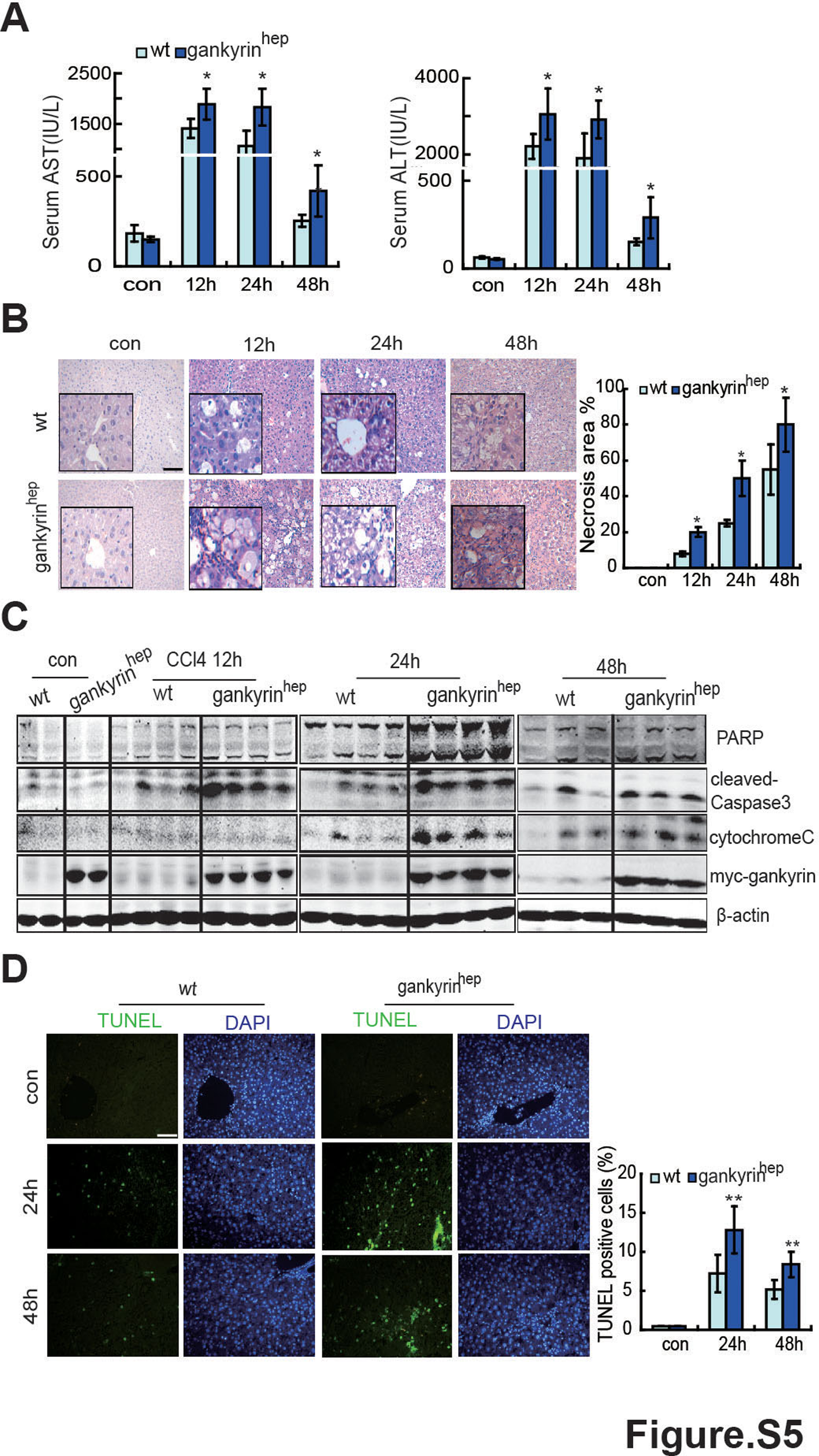

Supplement: Supplementary Figure 5 [file cddis2015120x6.tif]

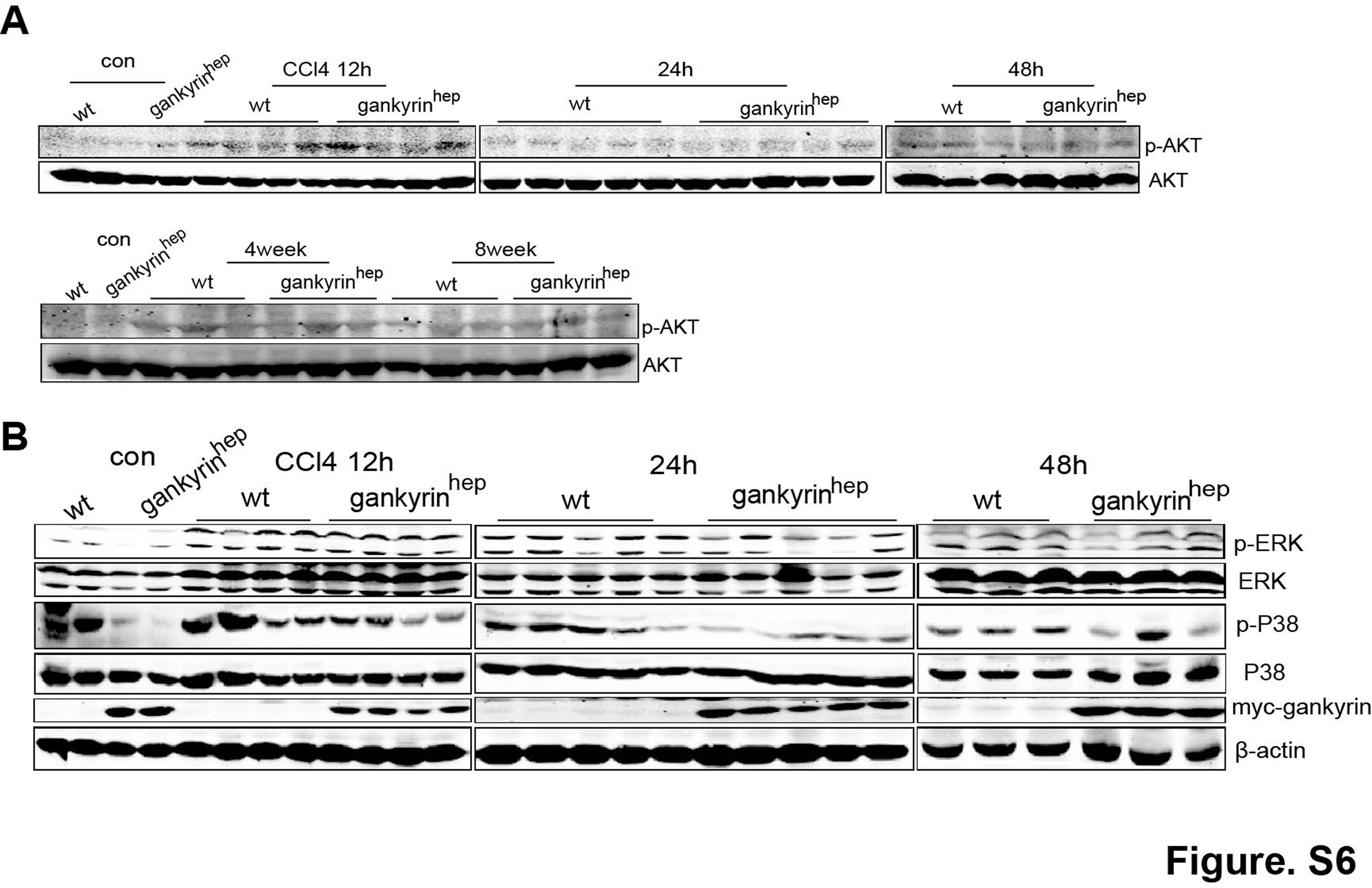

Supplement: Supplementary Figure 6 [file cddis2015120x7.tif]

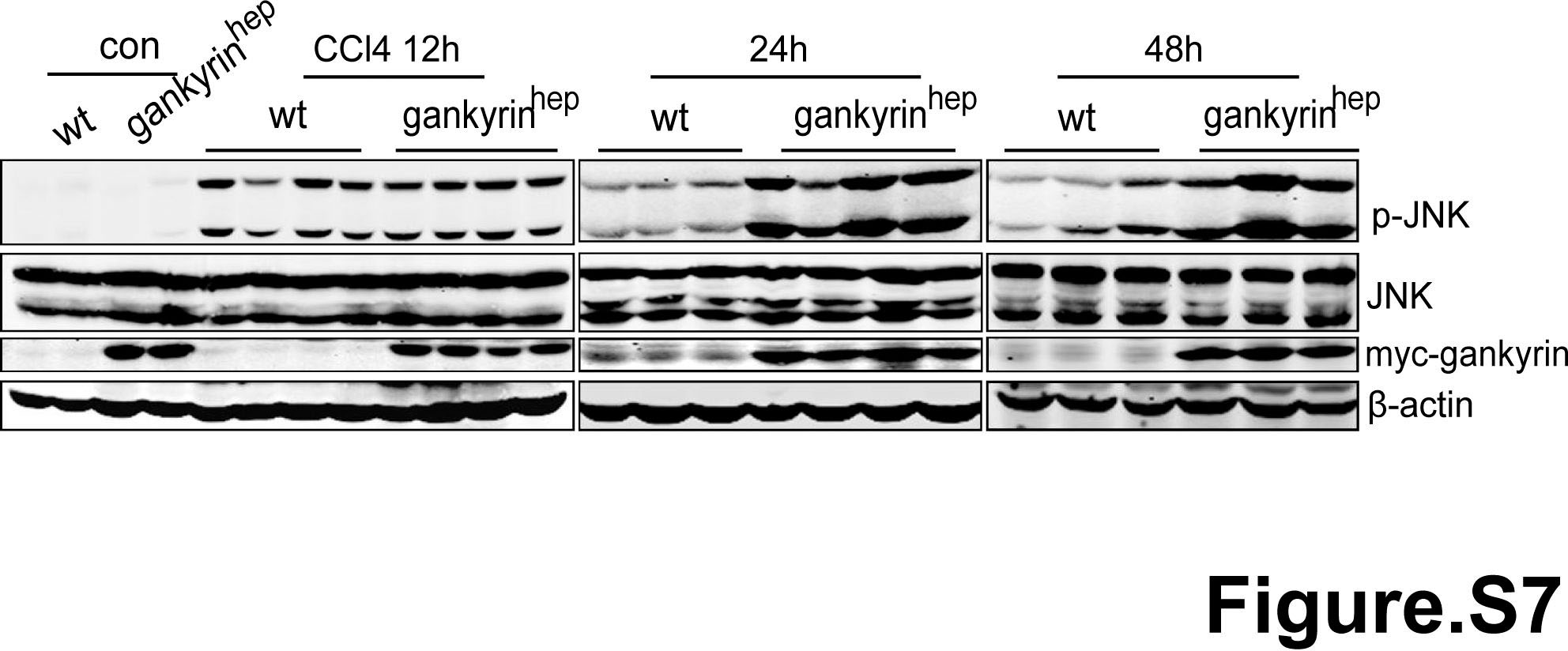

Supplement: Supplementary Figure 7 [file cddis2015120x8.tif]

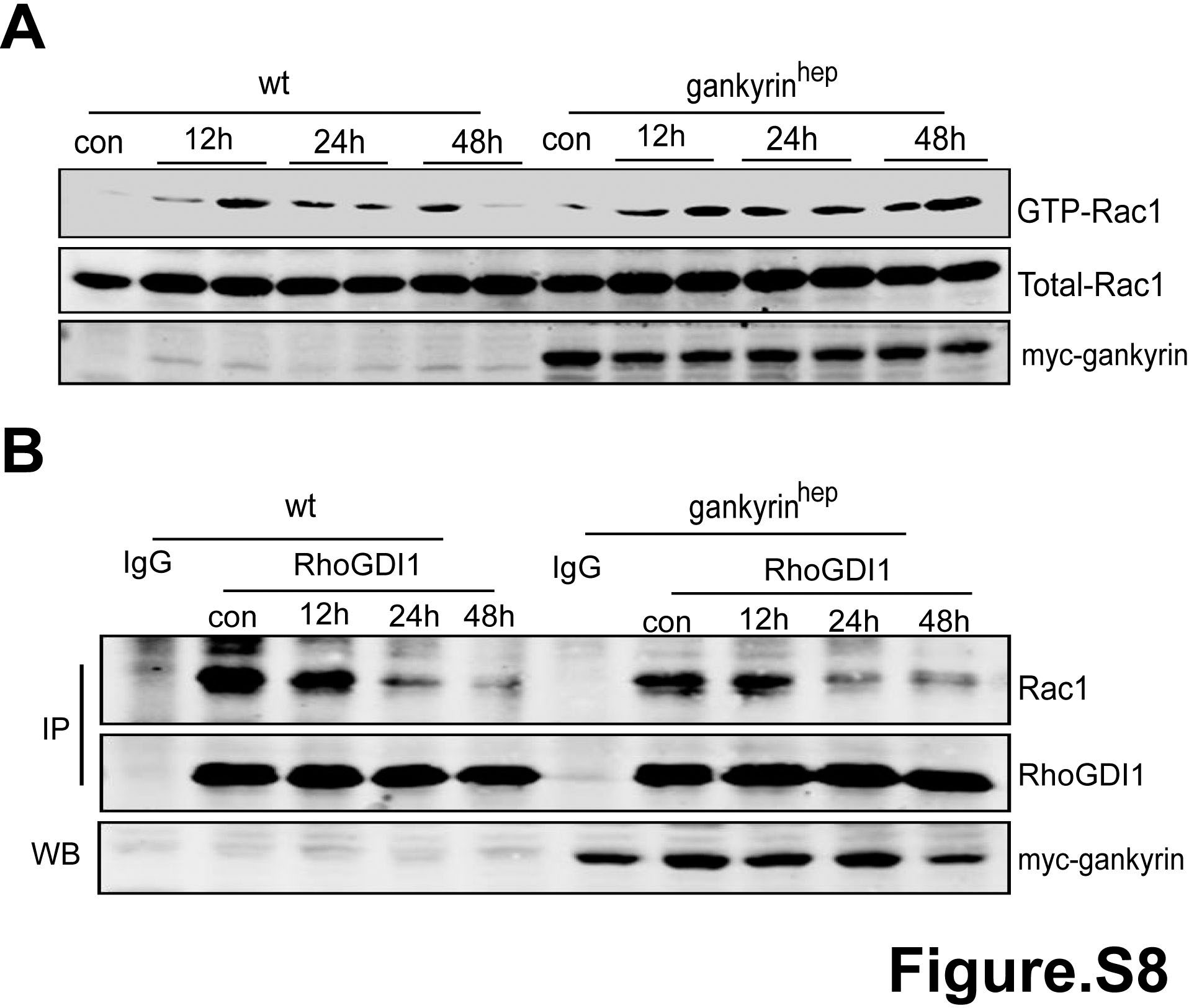

Supplement: Supplementary Figure 8 [file cddis2015120x9.tif]

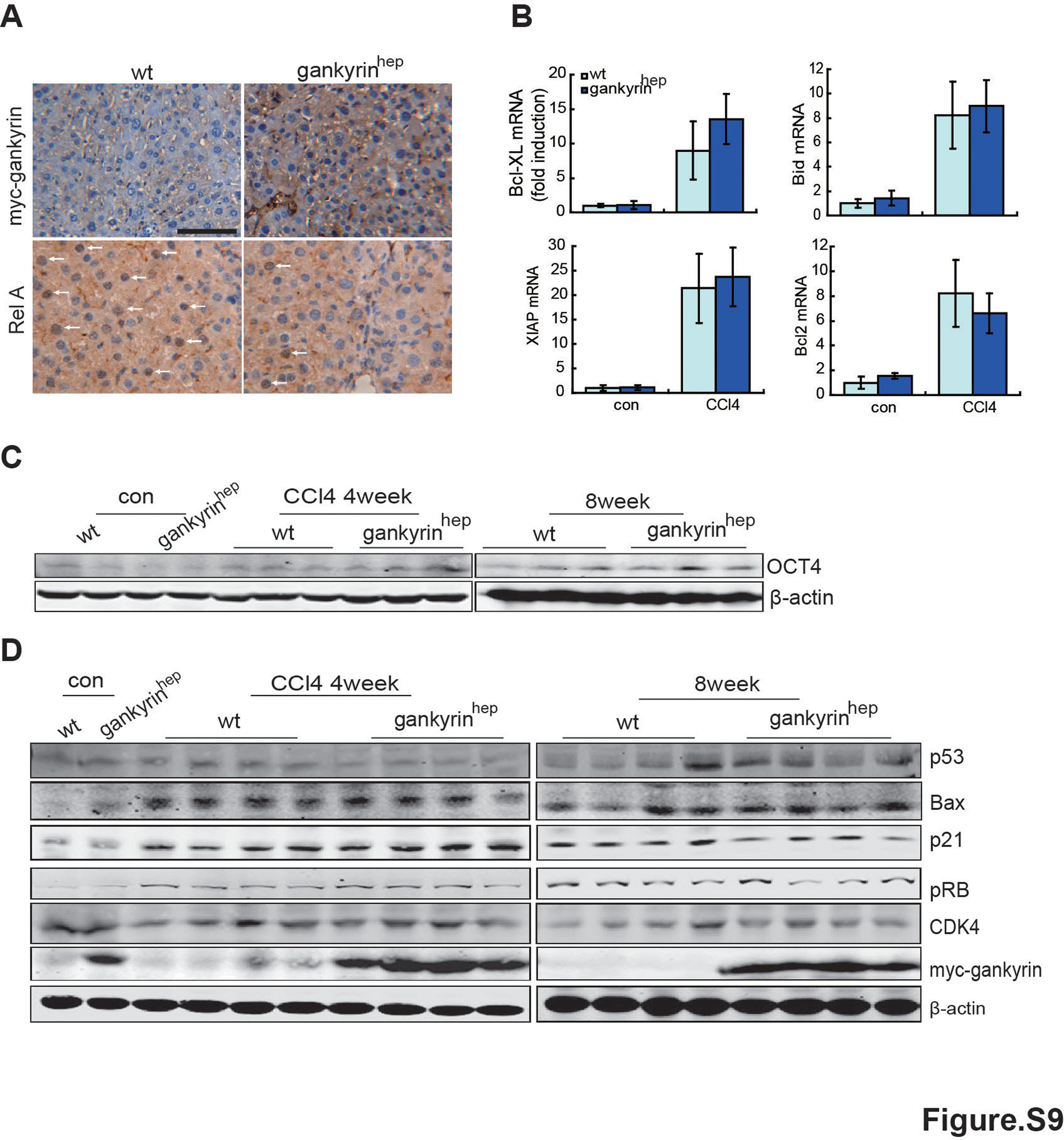

Supplement: Supplementary Figure 9 [file cddis2015120x10.tif]

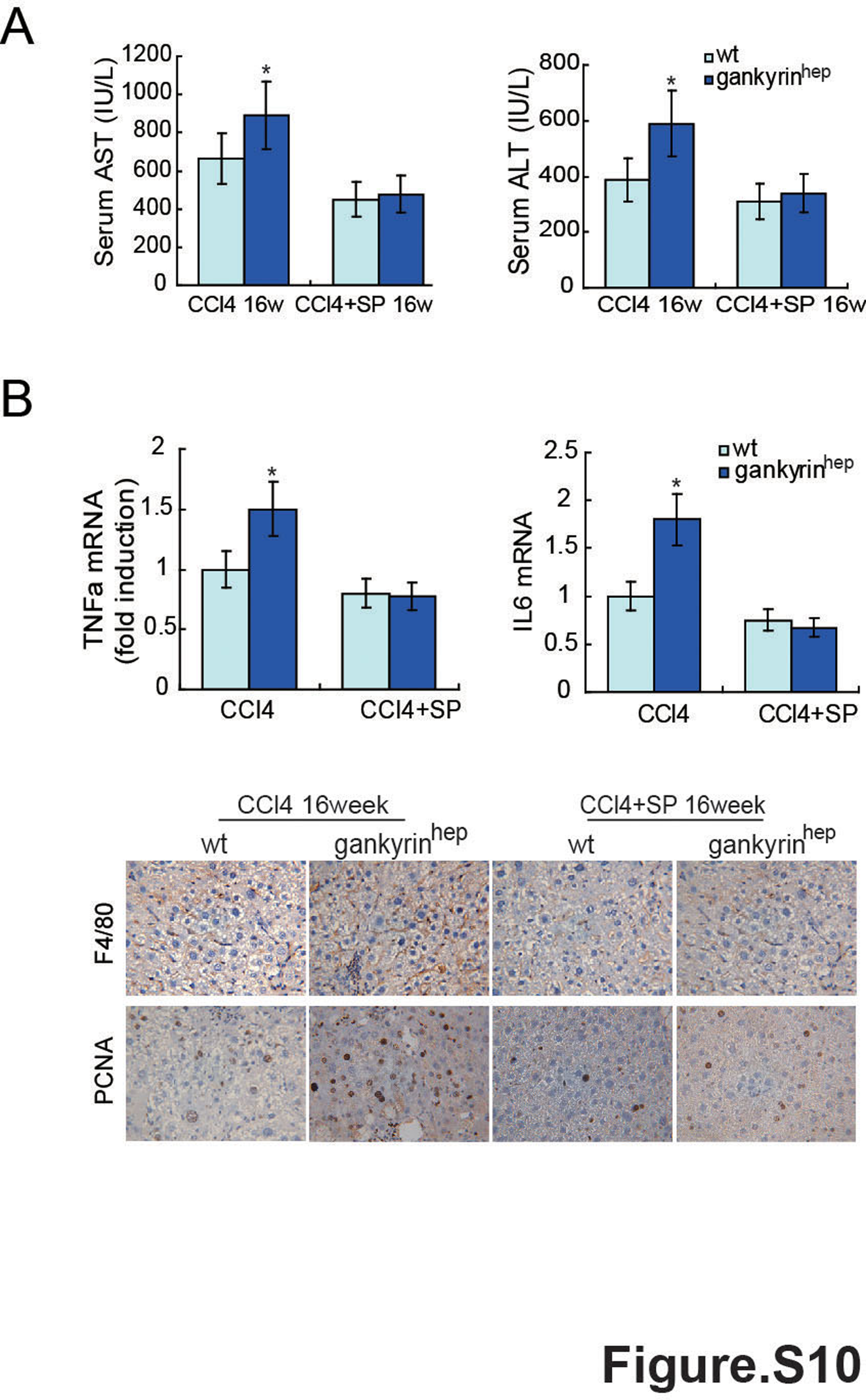

Supplement: Supplementary Figure 10 [file cddis2015120x11.tif]
